# Identification and validation of a novel necroptosis-related prognostic signature in cervical squamous cell carcinoma and endocervical adenocarcinoma

**DOI:** 10.3389/fonc.2022.1011000

**Published:** 2022-09-16

**Authors:** Weiyu Zhang, Wujun Cao, Zhuting Tong, Qinqin Jin, Xiya Jiang, Yinting Yang, Hui Yao, Guo Chen, Wei Gao, Yuting Zhu, Shuguang Zhou

**Affiliations:** ^1^ Department of Gynecology, Anhui Medical University Affiliated Maternity and Child Healthcare Hospital, Hefei, China; ^2^ Department of Gynecology, Anhui Province Maternity and Child Healthcare Hospital, Hefei, China; ^3^ Department of Clinical Laboratory, Anhui Province Maternity and Child Healthcare Hospital, Hefei, China; ^4^ Department of Radiation Oncology, The First Affiliated Hospital of Anhui Medical University, Hefei, China

**Keywords:** long noncoding RNA (lncRNA), necroptosis-related lncRNAs (NRLs), prognostic signature, immune, therapeutic agents, cervical squamous cell carcinoma and endocervical adenocarcinoma (CESC)

## Abstract

**Background:**

The purpose of this study was to investigate the prognostic signature of necroptosis-related lncRNAs (NRLs) and explore their association with immune-related functions and sensitivity of the therapeutic drug in cervical squamous cell carcinoma and endocervical adenocarcinoma (CESC).

**Methods:**

UCSC Xena provided lncRNA sequencing and clinical data about CESC, and a necroptosis gene list was obtained from the KEGG database. NRLs were selected by structuring a co-expression network of lncRNAs and necroptosis-related genes. To further screen lncRNAs, we used the univariate Cox regression method, Lasso regression, and multivariate Cox regression. Afterward, an NRL signature was established. We used the xCell algorithm and single-sample gene set enrichment analysis (ssGSEA) to clarify the pertinence between immune infiltration and NRL expressions in CESC patients and explored the relationship between the target lncRNAs and immune-related genes. By leveraging the GDSC database, the therapy-sensitive response of the prognostic signature was forecasted and an experimental validation was performed. We performed GSEA with the aim of recognizing the potential pathway related to the individual prognostic signature.

**Results:**

The two prognostic NRLs (AC009095.1 and AC005332.4) showed significant diversity and constituted the NRL signature. On the grounds of our signature, risk score was an independent element which was bound up with patient outcome (HR = 4.97 CI: 1.87–13.2, P = 0.001). The CESC patients were classified by the median risk score. Immune infiltration analysis revealed significant increases in CD4 + Tcm, eosinophils, epithelial cells, fibroblasts, NKT, plasma cells, platelets, and smooth muscle in the high-risk group (P< 0.05). Target lncRNAs also showed some correlation with NRGs. The estimated IC50 values of bicalutamide, CHIR.99021, and imatinib were lower in the high-risk group. Through the subsequent experimental validation, both AC009095.1 and AC005332.4 were significantly more highly expressed in SiHa than in Hela. AC009095.1 was expressed more highly in SiHa than in HUCEC, but the expression of AC005332.4 was reversed.

**Conclusions:**

This study elucidated that NRLs, as a novel signature, were indispensable factors which can significantly influence the prognosis of patients with CESC and could provide novel clinical evidence to serve as a potential molecular biomarker for future therapeutic targets.

## Introduction

Cervical squamous cell carcinoma and endocervical adenocarcinoma (CESC) have become two of the most leading cancer types in gynecology all over the world. With the invention of the human papillomavirus (HPV) vaccine, widespread adoption of CESC screening, and advancement in integrated therapy, the incidence of CESC exhibits a downward trend (1). However, even so, CESC continues to be a severe health problem among women and the mortality rates increase worldwide each year (2). Because of the deficiency of specificity and sensitivity, carbohydrate antigen 125 (CA125) and squamous cell carcinoma antigen, which belong to common clinical serum tumor biomarkers, were limited applications in clinical practice. In order to reduce morbidity and improve prognosis, we need neoteric, trustworthy, effective, and non-invasive tumor biomarkers (3).

Necroptosis, as a mode of programmed cell death, is triggered by RIPK1, RIPK3, and MLKL (4). Necroptosis was reported to participate in oncogenesis, cancer immunity, and metastasis (5). In CESC, RETRA (REactivation of Transcriptional Reporter Activity) induces necroptosis and increases ROS production (6), while RIPK3 expression is necessary for PolyIC-induced necroptosis (7). In addition, the low necroptosis process may predict poor prognosis in HPV-positive cervical cancers (8).

Long non-coding RNA (lncRNA) is one of numerous RNAs. Most lncRNAs do not participate in the protein translation process but take part in regulation of the gene expression at the transcriptional or posttranscriptional level (9). There are studies that have detected a close relation between necroptosis and lncRNAs (10). Necroptosis-related factors are involved in the ischemia-reperfusion process, and their main role is the regulation of programmed necrosis and myocardial injury (11). More recently, necroptosis-related lncRNAs (NRLs) have been extensively explored in predicting prognosis and immunotherapy response in breast cancer (12), colon cancer (13), gastric cancer (14), lung adenocarcinoma (15), and stomach adenocarcinoma (16). However, in fact, it remains to elucidate the potential role of NRLs in CESC.

In our research, we identified necroptosis-related lncRNAs of CESC and developed a risk model, with hopes of contributing helpful insights into the prognostic prediction and potential drug selection of CESC.

## Methods and materials

### Sample and data acquisition

UCSC Xena (http://xena.ucsc.edu/) provided the RNA sequencing (RNA-seq) data about CESC. The expression of normalized genes was detected as a single per million mapped reads per kilobase transcript fragment and log^2^-based transformation. The inclusion standard was listed as follows: (1) patients diagnosed with CESC; (2) patients with integrated lncRNA data and clinic information. On the basis of the inclusion criteria, 296 patients diagnosed with CESC were incorporated. In addition to that, TCGA database provided integrated clinic information for the patients. When filtrating clinic information, samples were abandoned which were less than 30 days of follow-up. The approval from the ethics committee was not required because TCGA database supplied all clinical data related to this study and strictly adhered to the publication guidelines of TCGA database (http://cancergenome.nih.gov/abouttcga/policies/publicationguidelines).

### Extraction of NRGs and lncRNAs

All data for lncRNAs were obtained from the RNA-seq data. Moreover, log2 transformation was utilized to normalize the total RNA expression data. The necroptosis gene list was obtained from the Kyoto Encyclopedia of Genes and Genomes (KEGG, https://www.genome.jp/kegg). Furthermore, the GENCODE (https://www.gencodegenes.org/human/release_23.html) database provided NRG information. The pertinence between lncRNAs and NRGs was determined by the Pearson correlation method. The lncRNAs relevant to necroptosis are the square of correlation coefficient|R^2^|> 0.5 and P< 0.001.

### Structure of the prognostic signature belonging to the NRLs

First of all, the prognostic value was evaluated by univariate Cox regression. Least Absolute Shrinkage and Selection Operator (Lasso) regression was applied to test the NRLs with P< 0.01 from the univariate analysis results. After that, the genes that were screened out by LASSO regression were admitted to a multivariate Cox model to calculate risk scores. We also calculated the risk model calculated as follows:


risk scores=∑(βi × Expi)

in which β_i_ refers to the coefficients indicating the weight of each signature and Exp_i_ indicates the expression of each signature. The patients meeting the inclusion criteria were classified on the basis of the median risk score. The log-rank statistical test was exploited to contrast the survival differences.

### Validation of prognostic signature

The individual prognostic signature model was built to validate prognostic features by adopting the Cox regression method. Time-dependent ROC curves were utilized to appraise the efficacy of our signature for predicting prognostic features. Moreover, these methods, which included decision curve analysis (DCA) and calibration curves, were applied to make a thorough inquiry into the accuracy of the signature model. Beyond that, we included demographic data and risk scores into the multivariate Cox regression and tested if they were independent elements which were bound up with patients’ prognosis.

### Extrapolation of immune-infiltrating cells in CESC

We exploited the R package “xCell” and single-sample gene set enrichment analysis (ssGSEA) with the aim of quantifying the abundance of immune cells in CESC patients. GSEA is a gene set-based enrichment analysis method which first determines the purpose of the analysis and then ranks based on the size of the association of the gene expression data and the phenotype (also understood as changes in expression). ssGSEA permits to define an enrichment score representing gene set absolute enrichment in each sample in a given dataset. ssGSEA was achieved by the R package “GSVA”, which estimated the integrated levels of immune cell types. xCell estimated the comprehensive levels of common immune cell types. xCell is an analytical approach on account of the gene signature, which integrates both the RNA-seq and microarray data and integrates the deconvolution approaches and advantages of the gene set enrichment. According to the ssGSEA and xCell instructions, gene expression profiles were prepared and the R package was run. At the same time, permutation was performed by using ssGSEA and xCell signatures. On the other hand, we used CAMOIP (17) (http://www.camoip.net) to analyze the association between target lncRNAs and immune-related genes with boxplots using the Mann–Whitney U test.

### Prediction of the sensitivity response to therapeutic agents

The sensitivity response to therapeutic agents of CESC patients was forecasted in the light of the data derived from Genomics of Drug Sensitivity in Cancer (GDSC; https://www.cancerrxgene.org). The half-maximal inhibitory concentration (IC50) was calculated through the R package “pRRophetic”.

### Gene set enrichment analysis

GSEA was exploited to discover the distinct enriched term with the aim of recognizing the potential pathway. By using the relevant database, CAMOIP (17) (http://www.camoip.net), the CESC patients grouped in line with the expression of the relevant lncRNA, then the enrichment analysis was performed.

### Cell lines

SiHa and Hela were human cervical cancer cell lines. SiHa was a cell line of cervical squamous cell carcinoma, and Hela was a cell line of cervical adenocarcinoma. They were used as the test group. HUCEC was a cell line of normal cervical, and PANC-1 was a cell line of pancreatic cancer. HUCEC cell lines were used as negative control groups and PANC-1 cell lines as a positive control group. All of them were obtained from Shanghai FuHeng Biotechnology company (Shanghai, China). We used DMEM contained with 10% FBS (Gibco, Grand Island, NY) to incubate SiHa and Hela cells. Then, we used RPMI 1640 supplementing 10% FBS (Gibco, Grand Island, NY) to incubate HUCEC and PANC-1 cells. Cells are cultured in an incubator at 37°C with 5% CO_2_.

### Quantitative real-time polymerase chain reaction

We used TRIzol Reagent (Invitrogen, Carlsbad, CA) to collect and lyse cells. Then, RNA cDNA first-strand synthesis kit (TransGen Biotech, Beijing, China) was utilized to obtain cDNA. Real‐time PCR was performed with One Step RT‐qPCR Kit (Sangon Biotech, Shanghai, China), and quantitative real-time polymerase chain reaction (qRT-PCR) was carried out as follows: 95°C for 3 min, and then 45 cycles of 95°C for 7 s, 57°C for 10 s, and 72°C for 15 s. The internal reference was the glyceraldehyde-3-phosphate dehydrogenase (GAPDH) gene. Information of primers is shown in **Table 1**.

**Table 1:** PCR primers used in this study.

Primer name	Primer type	Primer sequence (5′→3′)
**AC009095.1**	Forward	GAGAAAGGCESCTGCATAAGCG
	Reverse	GCESCTAATGGAACTCESCCTGCESC
**AC005332.4**	Forward	AATGCGAGGGCACATCAAGT
	Reverse	AGAGAGAGCGAGCGAGTGTA
**GAPDH**	Forward	GGAGCGAGATCESCCTCESCAAAAT
	Reverse	GGCTGTTGTCATACTTCTCATGG

### Statistical analysis

The survival curve was produced by means of the Kaplan–Meier method, which was detected by log-rank test. The effects, which were of necroptosis-related lncRNA signature and clinicopathological data on prognostic outcomes, were estimated by means of Cox regression and Lasso regression. This study’s statistical analysis was performed by adopting the R language (version 4.1.3 and version 4.2.0). Moreover, the bilateral test had statistical significance with P ≤ 0.01.

## Results

### Reconstruction of a co-expression network of NRGs and lncRNAs

We identified 18,016 lncRNAs in TCGA-CESC, and we obtained 159 genes related to necroptosis. In the NRGs, 133 genes were expressed in TCGA-CESC (**Table S1**). Furthermore, a lncRNA co-expression network relevant to NRGs was constructed with the aim of identifying the necroptosis-related lncRNAs. Finally, we selected 2,508 lncRNAs associated with necroptosis (∣R^2^∣ >0.5 and P< 0.001, **Table S2**).

### Appraisal of the prognostic signature relevant to NRLs

**Figure 1:**
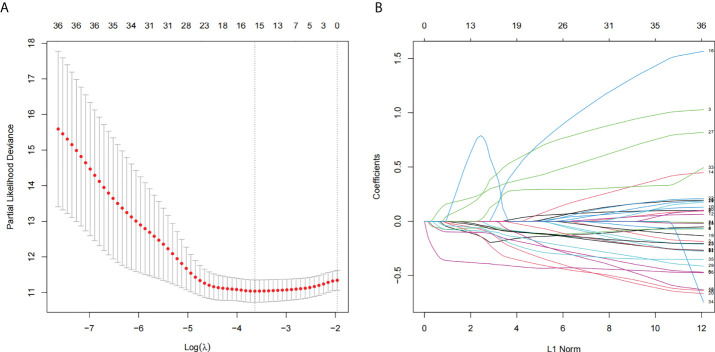
The lncRNAs related to necroptosis were screened by using the Lasso: **(A)** Lasso coefficient values for 36 NRLs in CSEC. The vertical dashed line is in the minimal log (lambda) value. **(B)** The overview of Lasso coefficients.

There were 36 NRLs meaningful for the patient outcome (P< 0.01, **Table S3**) after the univariate Cox analysis. After Lasso regression, 15 lncRNAs associated with necroptosis were filtrated (**Figures 1A, B**; **Table S4**). By using multivariate Cox regression analysis, AC009095.1 and AC005332.4 were discovered to be independent prognostic indicators. Amid two lncRNAs, there was a deleterious prognostic indicator which was named AC009095.1. On the other hand, AC005332.4 was a beneficial prognostic indicator (**Table 2**). Therefore, we took advantage of these two lncRNAs to set up a signature of NRLs. Moreover, we formulated the risk scores as hereunder mentioned: Risk score = (0.3857532*expression value of AC009095.1 - 0.3954274*expression value of AC005332.4).

**Table 2:** The results of lncRNAs on account of TCGA CESC data after the multivariate Cox regression.

lncRNA name	Gene name	coef	exp(coef)	se(coef)	Z score	Pr(>|z|)
**AC009095.1**	FTL	0.3857532	1.4707216	0.1812545	2.128241	0.03331709
**AC005332.4**	BCL2	-0.3954274	0.6733922	0.1690220	-2.339502	0.01930946

### The evaluation of the prognosis by the established signature

**Figure 2:**
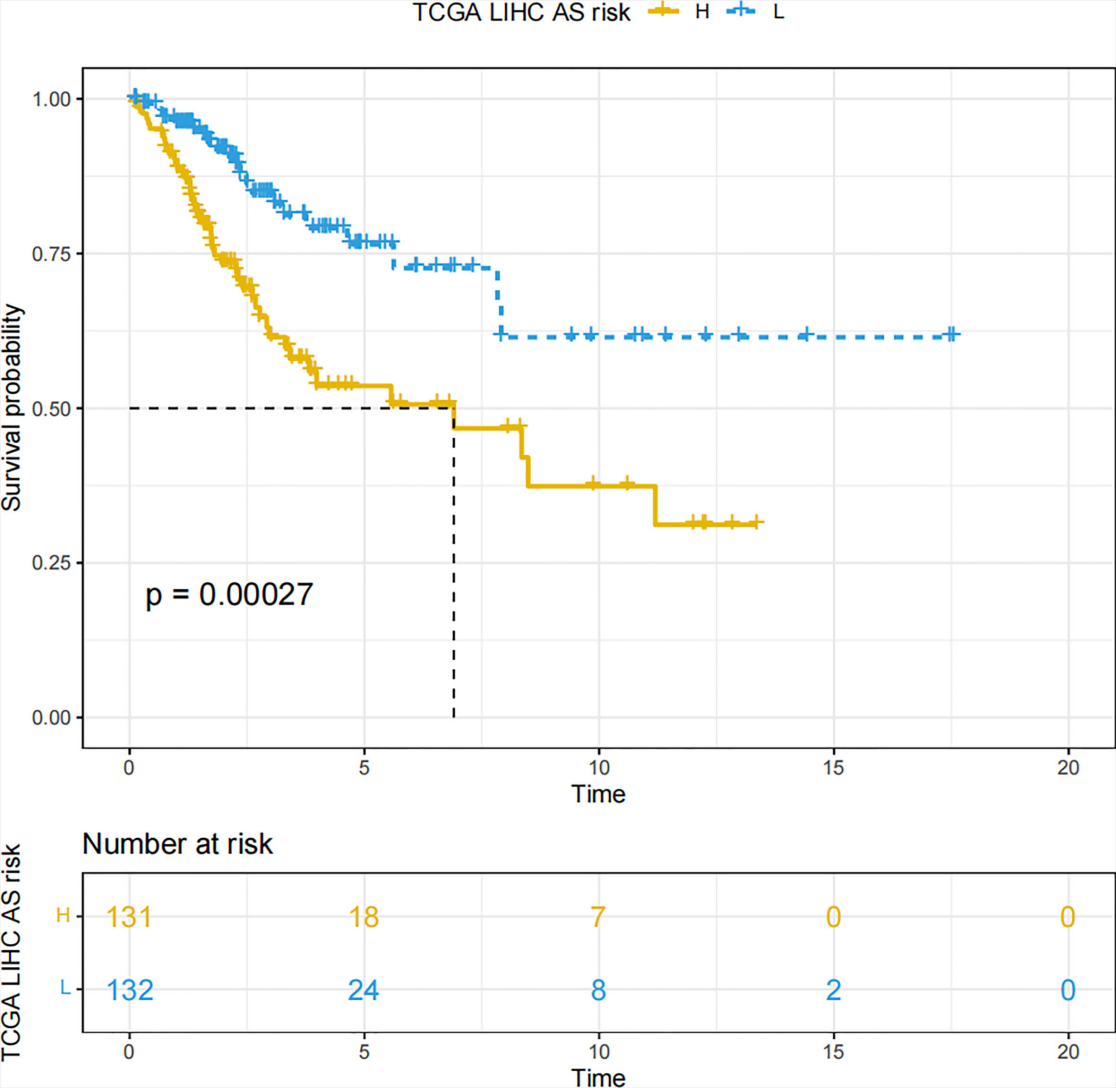
The Kaplan–Meier survival curves which are on account of the two NRLs’ risk scores.

**Figure 3:**
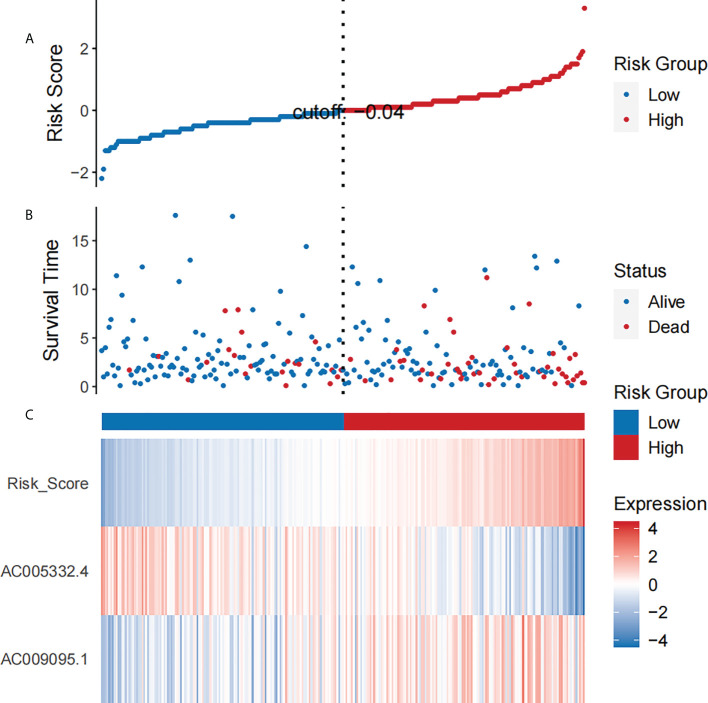
The analysis results which belong to CESC patients’ NRL signature. **(A)** The risk scores which belong to two groups. **(B)** The CESC patients’ survival time. **(C)** Heat map of the expression of the two NRLs and risk scores. The upward trend from low to high levels respectively corresponds to the colors which are from green to red.

By means of the analysis of the survival curves, we could conclude that risk scores were observably relevant to overall survival (OS). Moreover, the high-risk group had shorter OS (P< 0.001, log-rank test) (**Figure 2**). Meanwhile, Cox regression results pointed that risk scores had pivotal differences between the two groups (**Figure 3**).

### Clinical application of the signature relevant to necroptosis-related lncRNAs

**Figure 4:**
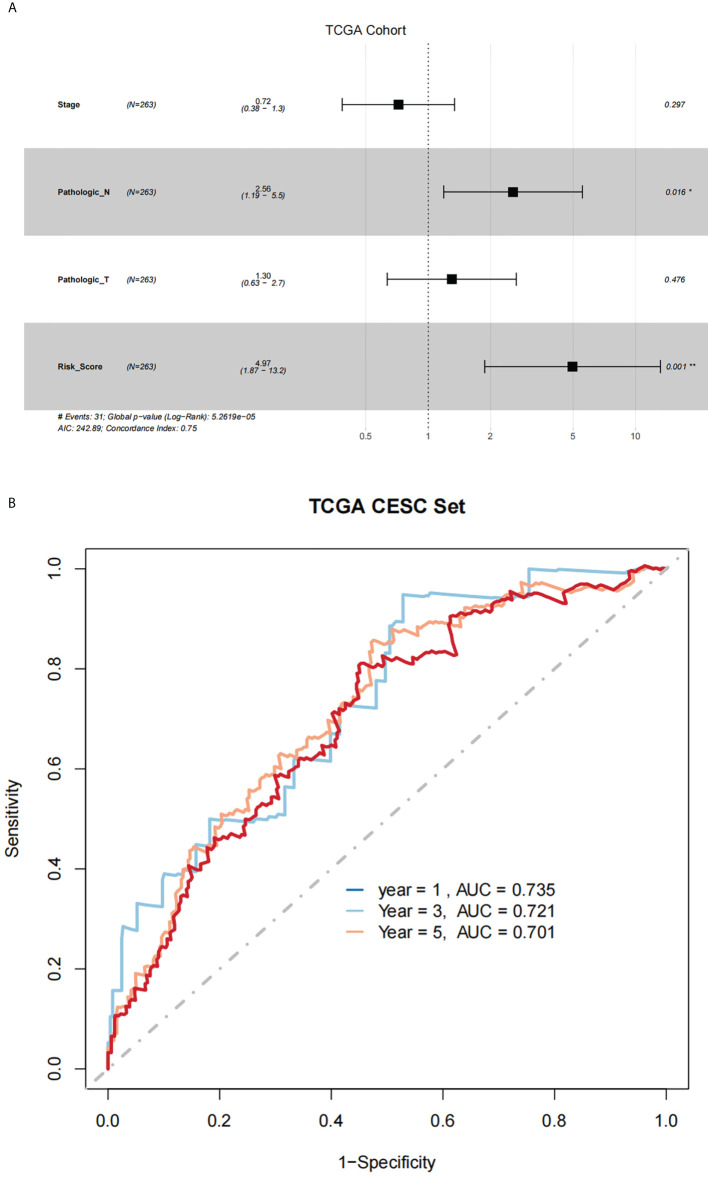
There was great predictive performance of the prognostic indicators on the basis of NRLs. **(A)** The forest plots which represented the results of the multivariate Cox regression analysis in CESC. **(B)** The areas were respectively 0.735, 0.721, and 0.701, which were under the ROC curves corresponding to 1, 3, and 5 years of survival.

**Figure 5:**
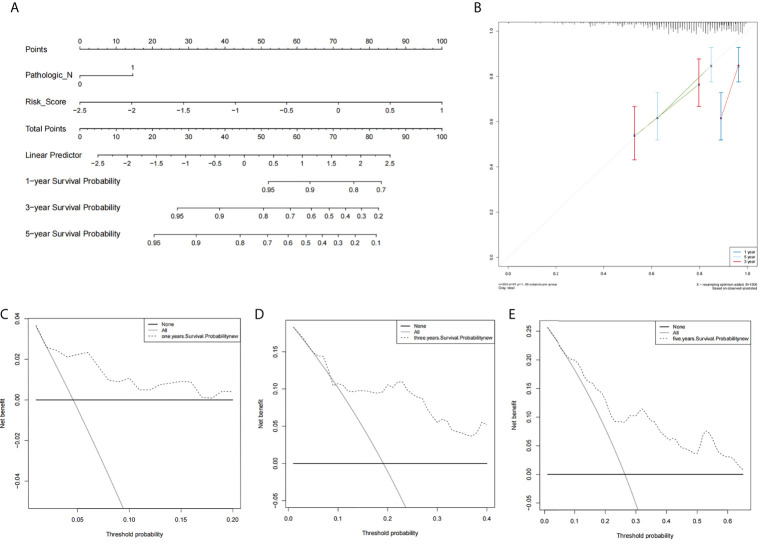
The prognostic assessment models in accordance with two lncRNAs related to the necroptosis. **(A)** The nomogram which consisted of 1-, 3-, and 5-year OS on the basis of the Pathologic_N stage and risk score. **(B)** The nomogram which predicted the probability of 1-, 3-, or 5-year survival and the calibration plots which were utilized to estimate the consistency between the predictions of the prognostic models and the actual OS. The 45° reference line expresses ideal calibration, in which the predicted probabilities are in accordance with the realistic probabilities. The decision curve analysis (DCA) of 1-year **(C)**, 3-year **(D)**, and 5-year **(E)** overall survival.

Using results from the multivariate Cox regression, it was not difficult to conclude that the Pathologic_N stage and risk score were isolated elements determining prognosis, which were with a HR risk score of 4.97 (95% CI: 1.87–13.2, P = 0.001, **Figure 4A**). Meanwhile, the areas under the ROC curves were respectively 0.735, 0.721, and 0.701, which corresponded to 1, 3, and 5 years of existence (**Figure 4B**). Beyond these, we also made the nomogram consisting of Pathologic_N staging and risk score. Also, Pathologic_N staging and risk score had the greatest effect on OS of 1, 3, and 5 years for patients with CESC as exhibited in the nomogram (**Figure 5A**). Furthermore, in comparison with the perfect pattern in the whole cohort, the calibration charts were well forecasted (**Figure 5B**). The results of DCA of three diverse survival rates also demonstrated that the nomogram had high potential for clinical application (**Figures 5C–E**).

### Immune cell type expression between two groups

**Figure 6:**
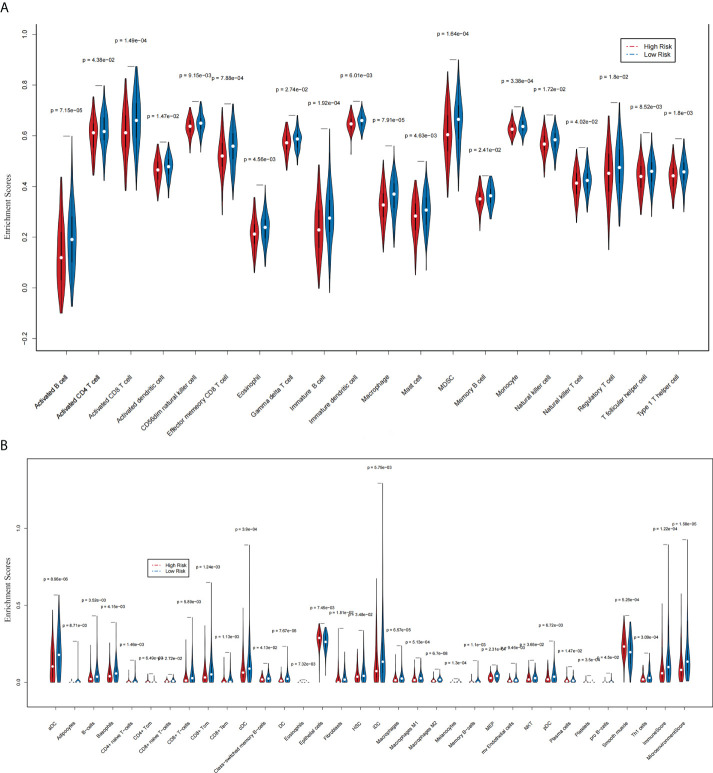
The association between two prognostic NRLs and immune infiltration (xCell and ssGSEA). **(A)** The violin plot which revealed that the immune cell expressions were different in two groups *via* ssGSEA. **(B)** The violin plot which revealed that the immune cell expressions were different in two groups *via* the xCell algorithm.

The ssGSEA results indicated that 20 immune cells were different in two groups, and all had a higher expression in the group with lower risk scores (**Figure 6A**). The xCell algorithm results revealed that 32 immune cells were different and the expression of eight immune cells were higher in the group with higher risk scores. They were respectively CD4^+^ Tcm (p = 6.49 e-03), eosinophils (p = 7.32 e-03), epithelial cells (p = 7.45 e-03), fibroblasts (p = 1.81 e-02), NKT (p = 3.65 e-02), plasma cells (p = 1.47 e-02), platelets (p = 3.5 e-04), and smooth muscle (p = 5.26 e-04) (**Figure 6B**).

### The expression of immune-related genes in the high- and low-expression groups of target lncRNAs

**Figure 7:**
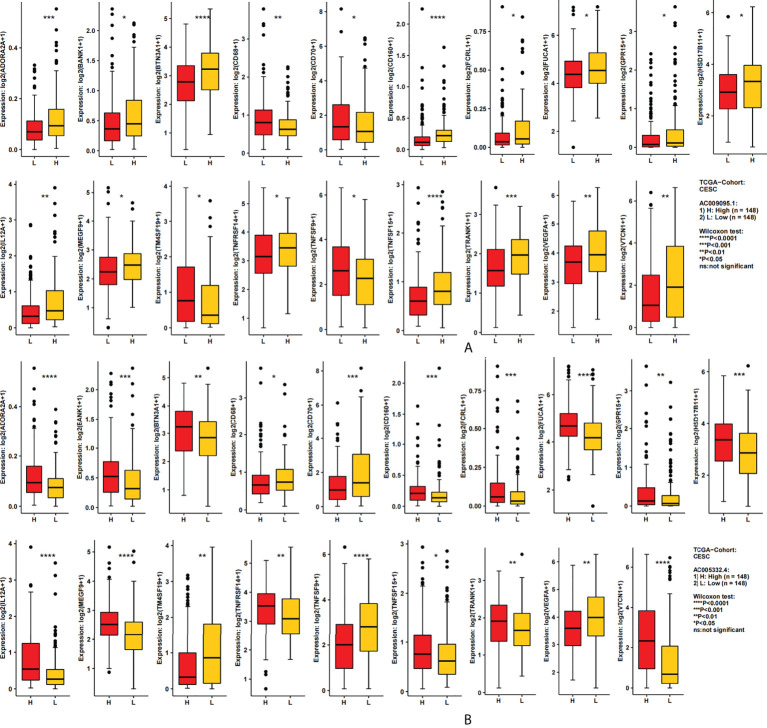
The relation between the target lncRNAs and the immune-related genes. **(A)** Expression of 19 immune-related genes in the AC009095.1 groups. **(B)** Expression of 19 immune-related genes in the AC005332.4 groups. *p < 0.05, **p < 0.01, ***p < 0.001, ****p < 0.0001.

We used the CAMOIP database to analyze the expression of immune-related genes in different groups of two lncRNAs. After screening, 19 immune-related genes were statistically differently expressed in different groups of two lncRNAs (**Figure 7A**, **Figure 7B**). These immune genes were ADORA2A, BANKI, BTN3A1, CD68, CD70, CD160, FCRL1, FUCA1, GPR15, HSD17B11, IL12A, MEGF9, TM4SF19, TNFRSF14, TNFSF9, TNFSF15, TRANK1, VEGFA, and VTCN1. It was found that CD68, CD160, TM4SF19, and TNFSF15 were all expressed more highly in the low expression group of two lncRNAs. However, for VEGFA in AC009095.1 groups, the expression was higher in the high expression group, and in AC005332.4 groups, the expression was higher in the low expression group. The remaining immune-related genes were all more highly expressed in the high expression group of two lncRNAs.

### The prediction of the signature for response to therapeutic agents

**Figure 8:**
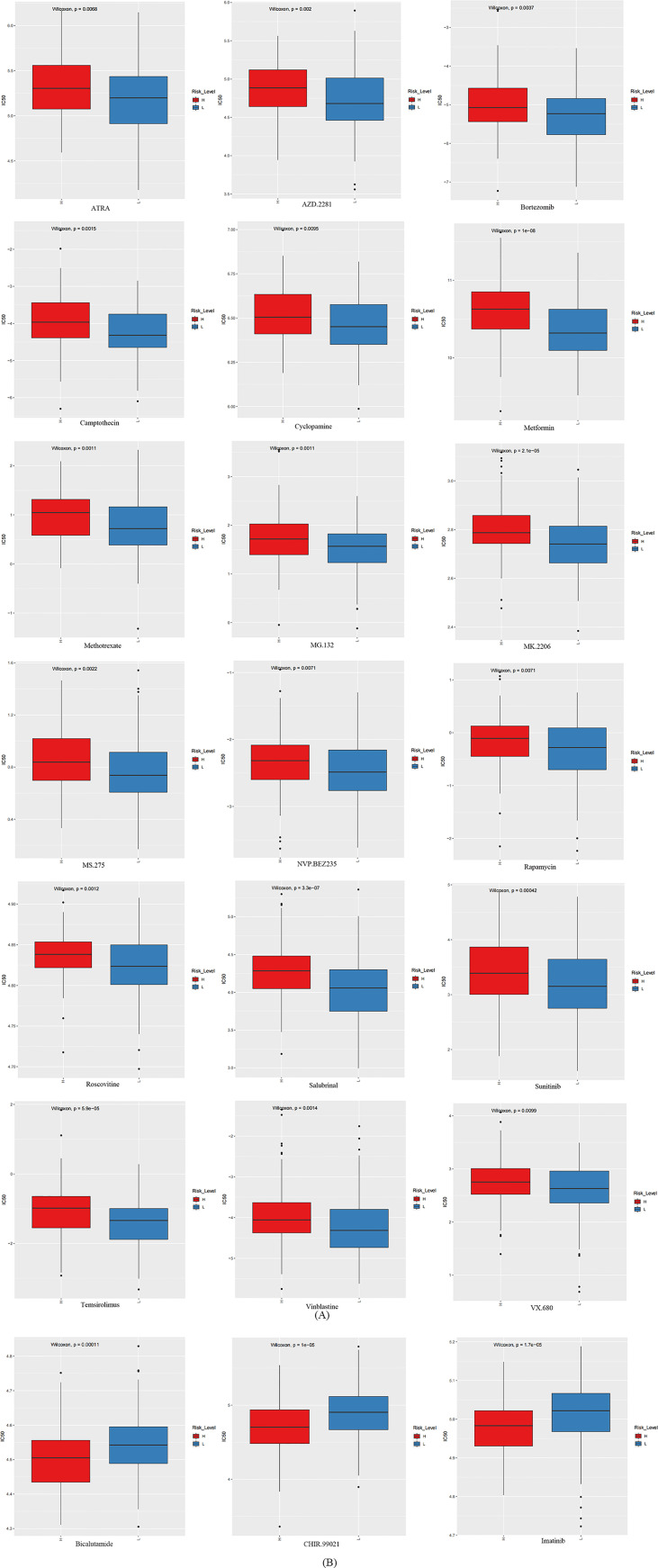
The box plots which revealed the IC50 values of 21 therapeutic agents between two groups. **(A)** The estimated IC50 values of 18 therapeutic agents were higher in the group with higher risk scores. **(B)** The estimated IC50 values of three therapeutic agents were higher in the group with lower risk scores.

The GDSC database was utilized to foretell the sensitivity reaction to therapeutic agents of the prognostic signature to frequently used therapeutic agents. Between the two groups, there were 21 therapeutic agents that varied significantly in IC50. The estimated IC50 values of 18 therapeutic agents were higher in the high-risk group (**Figure 8A**), which incorporated ATRA (p = 6.760884 e-03), AZD.2281 (p = 2.005571 e-03), bortezomib (p = 3.735047 e-03), camptothecin (p = 1.488292 e-03), cyclopamine (p = 9.461664 e-03), metformin (p = 1.010847 e-08), methotrexate (p = 1.084060 e-03), MG.132 (p = 1.134804 e-03), MK.2206 (p = 2.074208 e-05), MS.275 (p = 2.164303 e-03), NVP.BEZ235 (p = 7.133138 e-03), rapamycin (p = 7.098552 e-03), roscovitine (p = 1.194509 e-03), salubrinal (p = 3.341819 e-07), sunitinib (p = 4.200432 e-04), temsirolimus (p = 5.940194 e-05), vinblastine (p = 1.407201 e-03), and VX.680 (p = 9.917475 e-03). On the contrary, the estimated IC50 values of bicalutamide (p=1.063005 e-04), CHIR.99021 (p=1.045556 e-05), and imatinib (p=1.654762 e-05) were lower in the high-risk group (**Figure 8B**). This implied that bicalutamide, CHIR.99021, and imatinib had stronger sensitivity to patients with higher risk scores. The remaining therapeutic agents indicated stronger sensitivity to patients with lower risk scores.

### Results of the GSEA

**Figure 9:**
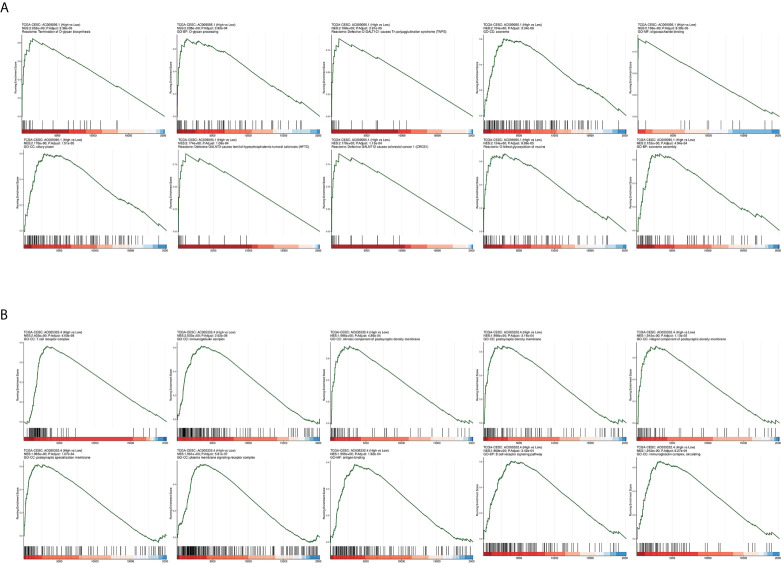
Results of GSEA in different groupings of the target lncRNAs in CESC from TCGA database. **(A)** Results of GSEA in groups of AC009095.1. **(B)** Results of GSEA in groups of AC005332.4.

**Figure 10:**
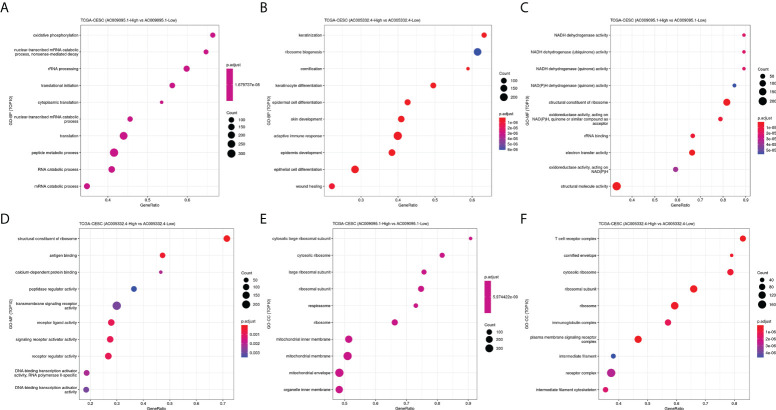
Results of GO-BP, GO-MF, and GO-CC. **(A)** Results of GO-BP in groups of AC009095.1. **(B)** Results of GO-BP in groups of AC005332.4. **(C)** Results of GO-MF in groups of AC009095.1. **(D)** Results of GO-MF in groups of AC005332.4. **(E)** Results of GO-CC in groups of AC009095.1. **(F)** Results of GO-CC in groups of AC005332.4.

The CESC patients were grouped according to lncRNA expression levels in the CAMOIP database. We performed GSEA and obtained the following results. The top 10 pathways with the enrichment scores in the results of enrichment analysis regarding AC009095.1 are respectively termination of O-glycan biosynthesis, O-glycan processing, defective C1GALT1C1-caused Tn polyagglutination syndrome (TNPS), axoneme, oligosaccharide binding, ciliary plasm, defective GALNT3-caused familial hyperphosphatemic tumoral calcinosis (HFTC), defective GALNT12 causes colorectal cancer 1 (CRCS1), O-linked glycosylation of mucins, and axoneme assembly (**Figure 9A**). The top 10 pathways with the enrichment scores in the results of enrichment analysis regarding AC005332.4 are respectively T-cell receptor complex, immunoglobulin complex, intrinsic component of postsynaptic density membrane, postsynaptic density membrane, integral component of postsynaptic density membrane, postsynaptic specialization membrane, plasma membrane signaling receptor complex, antigen binding, B-cell receptor signaling pathway, and immunoglobulin complex or circulating (**Figure 9B**). Subsequently, enrichment analysis was performed again in line with three aspects of functional enrichment analysis, cellular component (GO-CC), molecular function (GO-MF), and biological processes (GO-BP). In terms of biological processes, AC009095.1 was mainly enriched in the oxidative phosphorylation, nonsense-mediated decay, translational initiation, cytoplasmic translation, translation, peptide metabolic process, and the pathways related to RNA catabolism (**Figure 10A**). AC005332.4 was mainly enriched in keratinization, ribosome biogenesis, cornification, keratinocyte differentiation, epidermal cell differentiation, skin development, adaptive immune response, epidermis development, epithelial cell differentiation, and wound healing (**Figure 10B**). In terms of molecular function, AC009095.1 was mainly enriched in some of the related pathways of NADH dehydrogenase activity (**Figure 10C**). AC005332.4 was mainly enriched in the binding to the receptor-associated pathways (**Figure 10D**). In terms of cellular component, AC009095.1 was mainly enriched on the ribosome-associated pathways (**Figure 10E**). AC005332.4 was mainly enriched in the T-cell receptor complex, cornified envelope, cytosolic ribosome, ribosomal subunit, ribosome, immunoglobulin complex, receptor complex, and so on (**Figure 10F**).

### Results of qRT-PCR

**Figure 11:**
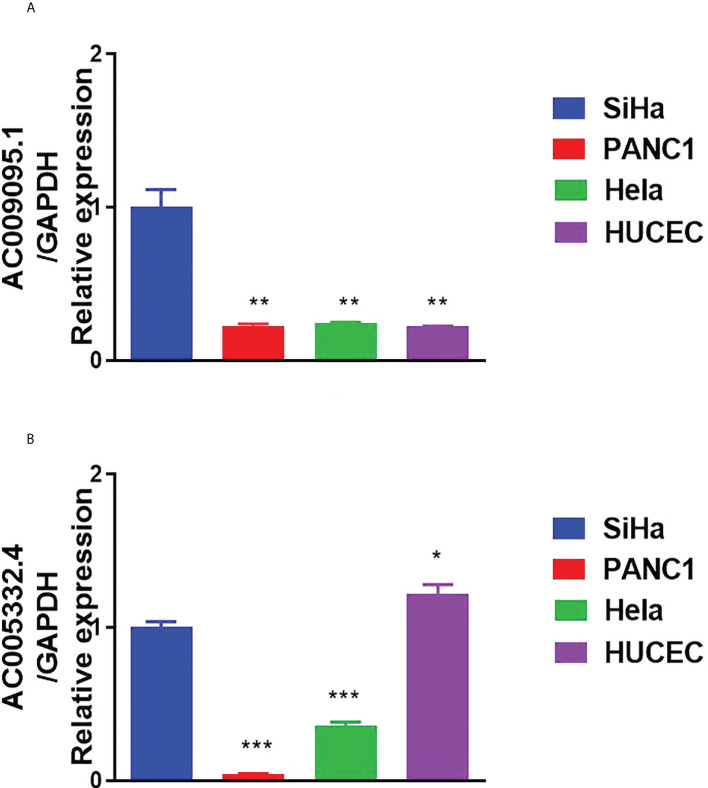
Results of qRT-PCR. **(A)** The expression of AC009095.1 in the four cell lines and AC009095.1 expression were significantly higher in SiHa cell lines than in the other three cell lines. **(B)** The expression of AC005332.4 in the four cell lines and AC005332.4 expression were higher in SiHa cell lines than in PANC-1 and Hela cell lines but were slightly lower than in HUCEC cell lines. *p < 0.05, **p < 0.01, ***p < 0.001.

We used qRT-PCR to detect the expression of the target lncRNAs in different cell lines. The experimental results indicated that AC009095.1 expression was significantly higher in SiHa cell lines than in the other three cell lines (**Figure 11A**). AC005332.4 expression was higher in SiHa cell lines than in PANC-1 and Hela cell lines but was slightly lower than in HUCEC cell lines (**Figure 11B**).

## Discussion

Besides breast, colorectal, and lung cancer, CESC is the most common cancer among women (18). In developed countries, the trend of the incidence and mortality of cervical cancer is declining (19), but in underdeveloped countries, it remains high (20). Furthermore, squamous cell carcinoma is the most common type of CESC (21), and only a small proportion of patients with cervical squamous cell carcinoma can be cured with conventional surgery, while a majority of patients develop tumor recurrence and advanced metastases (22–24).

Necroptosis participates in a variety of disease processes (25). Necroptosis has been reported to have different effects in different cancer types. As a mode of death in abnormal cells, necroptosis can abate tumor occurrence. At the same time, necroptosis can also trigger inflammatory responses and promote cancer metastasis as well as immunosuppression (26). Notably, there is little study which is involved in both lncRNA and necroptosis. For example, TRINGS (Tp53-regulated inhibitor of necrosis under glucose starvation) was mentioned to appear in cancer cells in two studies (27, 28). Another study mentioned that NRL Linc00176 could influence liver cancer cell survival and cell cycle (29).

For the purpose of better comprehending the roles of NRLs in the occurrence and development of CESC, first of all, we probed the associated expression of lncRNAs in patients, which were from TCGA database. After that, by using the Pearson analysis, we ascertained the co-expression connection between NRGs and lncRNAs and obtained 2,508 NRLs. Then, after the univariate Cox regression, 36 lncRNAs relevant to necroptosis with prognostic value were selected. After using the Lasso regression method, 15 lncRNAs relevant to necroptosis were identified. Subsequently, there were two NRLs found by further multivariate Cox regression analysis, which were as follows: AC009095.1 and AC005332.4. Next, we calculated the risk scores for the two lncRNAs relevant to necroptosis and established a signature of lncRNAs relevant to necroptosis. Simultaneously, the patients were grouped in terms of median risk score. Notably, the patients with higher risk scores showed worse prognosis. Moreover, in the high-risk group, AC009095.1 had a higher expression while the result of AC005332.4 was the opposite. Consistent with the above bioinformatic analysis, our experimental results of qRT-PCR demonstrated that AC009095.1 was more highly expressed in Hela and SiHa cell lines compared with PANC-1 (positive control) and HUCEC (negative control) cell lines. AC005332.4 was expressed more highly in both SiHa and Hela than in the positive control, but it was lower than in the negative controls. Therefore, it showed that the two lncRNAs had a vital role in the growth of CESC. Because both were more highly expressed in SiHa than in Hela, they may play a more positive role in the development of cervical squamous cell carcinoma. Relative to normal cervical cell lines, the expression of AC009095.1 was higher in cervical cancer cells, while AC005332.4 was the opposite. It was indicated that AC009095.1 may be involved in tumor occurrence and development; however, AC005332.4 may reduce tumor occurrence and development.

Besides, it was proved that our signature owned a favorable prognostic assessment effect in light of the univariate and multivariate Cox regression. Moreover the nomogram displayed that the Pathologic_N stage and risk score had the greatest effect on OS of CESC patients. The areas corresponding to three diverse survival rates were respectively 0.735, 0.721, and 0.701, which were under the ROC curve. This outcome suggested that the signature of risk score had some latent capacity in the aspect of predicting survival. Subsequently, we validated the accuracy of the ideal model according to the calibration curve. In the interest of further exploring the clinic application of the signature, this study investigated the correlation of the signature by DCA with clinic characteristics, and the consequences demonstrated that our nomogram had great potential for clinic application.

It is complex and difficult to interpret the interaction between tumors and their immune microenvironment, but it is importantly implicated in therapeutic strategies and the development of novel prognostic markers (30). This study clarified the relevance of the expression of lncRNAs relevant to necroptosis AC009095.1 and AC005332.4 and immune infiltration in CESC by using xCell package and ssGSEA. Most importantly, as a result of immune cell infiltration analysis, there was a negative relation between the risk score and aDC, adipocytes, B cells, basophils, CD8 ^+^ Tcm, CD8 ^+^ Tem, cDC, DC, HSC, iDC, melanocytes, and so on, whereas CD4 ^+^ Tcm, eosinophils, epithelial cells, fibroblasts, NKT, plasma cells, platelets, and smooth muscle were increased in the high-risk group. It is reported that B cells have anticancer effects in human papillomavirus-associated squamous SCC and have significant beneficial effects on patient prognosis (31). As preclinical evidence from Cao’s studies, DCs derived from monocyte activate T cells restrain tumor development by inhibiting tumor cell propagation and accelerating apoptosis. Moreover, tumor cell proliferation is inhibited by cytokines which are secreted by DCs and T cells (32). However, the higher the clinical stage of cervical cancer was, the lower the Th1 level was. It was indicated that Th1 could reduce the development of cervical cancer (33). In another aspect, mDCs and cervical fibroblasts instructed by cocultures of CESC enhanced the tumorigenicity of Th17 cells *in vitro* (34). The cytotoxicity of NKT cells is closely related to the development of cervical cancer. It was found that enhanced tolerance of NKT cells could promote cervical cancer progression (35). It was discovered that thrombocytosis, known as excessive platelets in the blood, was an independent prognostic element in cervical cancer (36). Furthermore, platelets took part in tumor cell extravasation, tumor growth, and metastasis (37). Eosinophils enhanced infiltration of CD8 ^+^ T cells and normalized tumor vessels to mediate tumor rejection in early animal studies (38). These were in general agreement with our findings. Based on the results of the immunoassay, we found that overall CD4 ^+^ T cells were expressed higher in the high-risk group, while CD8 ^+^ T cells were expressed higher in the low-risk group. Moreover, activated memory CD4 ^+^ T cells are considered an element of the favorable outcomes about patients with cervical squamous cell carcinoma, whereas resting memory CD4 ^+^ T cells are considered an element of the adverse outcomes (39). A study of triple-negative breast cancer (TNBC) showed that a high CD4 ^+^ Tcm enrichment score was associated with worse RFS of patients with TNBC (40). However, the specific role of CD4 ^+^ Tcm in cervical cancer is not currently clear. In early CESC, CD8 ^+^ T cells and CD8 ^+^/CD4 ^+^ ratio are apparently increased. Both overall survival and disease-free survival are reduced when the CD8 ^+^/CD4 ^+^ ratio is below 2, which have been reported in other research studies (41). Moreover, rapid tumor growth and lymph node metastasis are closely related to the reversion of the CD8 ^+^/CD4 ^+^ ratio in patients with cervical cancer (42). In a word, in early cervical cancer, the proportion of CD8 + T cells was higher than that of CD4 ^+^ T cells, and it played a relatively important role. When cancer developed further, the proportion of CD4 ^+^ T cells increased significantly. When the proportion of CD4^+^ T cells was higher than that of CD8 ^+^ T cells, it indicated that the cancer already had lymph node metastasis associated with immune infiltration. When the ratio gradually decreased, the likelihood of a poor patient prognosis was greater.

With the aim of exploring the relationship between the target lncRNAs and immune-related genes, we divided the target lncRNAs into different groups and analyzed the expression of immune-related genes. It was shown that 19 immune-related genes were statistically differently expressed in different groups. These immune genes were ADORA2A, BANKI, BTN3A1, CD68, CD70, CD160, FCRL1, FUCA1, GPR15, HSD17B11, IL12A, MEGF9, TM4SF19, TNFRSF14, TNFSF9, TNFSF15, TRANK1, VEGFA, and VTCN1. A study has shown that ADORA2A and VTCN1 have a certain relationship with the immune infiltration of CESC (43). In cervical cancer, BTN3A1 overexpression could inhibit the cervical cancer cell phenotype. In other words, BTN3A1 had inhibitory effects on the development of cervical cancer (44). In Ovestad’s study, CD160 was also downregulated in the CIN3/AIS lesions (45). The CD68 tumor-associated macrophages in CESC were significantly increased than those in normal tissue or paracarcinoma, and high stromal CD68 tumor-associated macrophages were born on lymph node metastasis (46). The upregulation of FUCA1 in SiHa is shown in Kalliopi’s study (47). The IL-12A gene was related to enhanced risk of cervical cancer (48). VEGFA was validated an unfavorable molecules in HPV cervical squamous cell carcinoma (49). The remaining immune-related genes have not been explored in cervical cancer. This is also our next research direction.

Overall, the necroptosis-related signature that we constructed, containing both AC009095.1 and AC005332.4, played a certain role in the immune function. In the immune infiltration analysis, most immune cells were highly infiltrated in the low-risk group. Moreover, the necroptosis-related signature involvement in immune infiltration is more likely achieved through CD4 ^+^ Tcm, eosinophils, epithelial cells, fibroblasts, NKT, plasma cells, platelets, and smooth muscle. Combined with the results of the enrichment analysis, we could conclude that the major immune role in the necroptosis-related signature is AC005332.4.

In the light of the analysis of the therapeutic agents to the signature we established, we could conclude that three therapeutic agents are more treatment sensitive to the low-risk group. Among them, imatinib is currently considered to own a vital effect in the treatment of CESC but even with paclitaxel more often (50). Bialutamide also has some therapeutic potential in tumors (51), but the therapeutic effect in CESC is still unknown. Thus, it can be seen that the signature we established as well as the prediction of therapeutic drugs have credible evidence.

Furthermore, in the existing studies, we have not found any reports involved in AC009095.1. Of note, the enrichment analysis results showed that AC009095.1 participates in the pathway, and defective GALNT12 causes colorectal cancer 1 (CRCS1), but there is no relevant evidence to support it. AC005332.4 is mostly enriched in pathways related to immunity, such as T-cell receptor complex, immunoglobulin complex, antigen binding, B-cell receptor signaling pathway, and immunoglobulin complex or circulating. This indicates that AC005332.4 is closely related to the immune function (52). Meanwhile, AC005332.4 has also been reported in CESC (52), colorectal cancer (53), breast cancer (54), and osteosarcoma (55).

Admittedly, our study has some limitations. First, we did not establish the co-expression network which probably existed in lncRNAs and mRNA. Moreover, the specific molecular mechanisms of the NRLs (AC009095.1 and AC005332.4) have not been verified specifically in the experiments. We also did not perform experiments to verify the selected drug sensitivity. Our sample size was not sufficient, and some of the analysis results may be precise.

By reason of the foregoing, we finally succeeded in constructing the risk score signature in the light of the two necroptosis-related lncRNAs, which was an independent prognostic element in CESC patients. Our study supplied a profound scientific insight of the function of necroptosis in biological traits of malignant tumors. It also advancedly proposed a double-necroptosis-related lncRNA signature that provides effective and valuable clinic applications for dependable prognostic prediction and individuation therapy of CESC patients. The proposed method improves the prediction accuracy of the target lncRNAs, and these lncRNAs relevant to necroptosis have important implications for prognosis and prediction of therapeutic markers in CESC patients. Therefore, the function of these necroptosis-related lncRNAs of CESC is encouraging enough to warrant advanced exploration.

## Data availability statement

Publicly available datasets were analyzed in this study. This data can be found here: The dataset for this study can be found in the UCSC Xena (http://xena.ucsc.edu/), TCGA (http://cancergenome.nih.gov/abouttcga/policies/publicationguidelines), KEGG (https://www.genome.jp/kegg), GENCODE(https://www.gencodegenes.org/human/release_23.html) and GDSC (https://www.cancerrxgene.org).

## Author contributions

SZ, GC, and ZT conceived, designed, and supervised the study. WZ, HY, WC, QJ, XJ, and YY drafted the manuscript and performed the data analysis and visualization. WG and YZ collected the data. All authors devoted to data interpretation, manuscript preparation, editing, and review.

## Funding

This work was supported by the Applied Medicine Research Project of Hefei Health Commission (Grant No. HWKJ2019-172-14), the Research Fund Project of Anhui Medical University (Grant No. 2020xkj236), and the Natural Science Foundation of Higher Education Institutions of Auhui Province (Grant No. KJ2021A0352).

## Acknowledgments

We acknowledge the UCSC Xena, TCGA, KEGG, GENCODE, and GDSC databases for providing their platforms and contributors for uploading their meaningful datasets.

## Conflict of interest

The authors declare that the research was conducted in the absence of any commercial or financial relationships that could be construed as a potential conflict of interest.

## Publisher’s note

All claims expressed in this article are solely those of the authors and do not necessarily represent those of their affiliated organizations, or those of the publisher, the editors and the reviewers. Any product that may be evaluated in this article, or claim that may be made by its manufacturer, is not guaranteed or endorsed by the publisher.

## Supplementary material

Supplementary Table 1 The gene list expressed in CESC related to necroptosis.

Supplementary Table 2 The necroptosis-related lncRNAs were screened by a co-expression network.

Supplementary Table 3 Univariate Cox results of necroptosis-related lncRNAs.

Supplementary Table 4 Lasso regression results of necroptosis-related lncRNAs.
